# A continuum model for the elongation and orientation of Von Willebrand factor with applications in arterial flow

**DOI:** 10.1007/s10237-024-01840-8

**Published:** 2024-04-09

**Authors:** E. F. Yeo, J. M. Oliver, N. Korin, S. L. Waters

**Affiliations:** 1 Department of Mathematics, University College London, London, UK; 2 Mathematical Institute, University of Oxford, Oxford, UK; 3 Biomedical Engineering, Technion, Haifa, Israel

**Keywords:** Mathematical model, Von Willebrand factor, Arterial thrombosis, Computational fluid dynamics, Parameter identification

## Abstract

The blood protein Von Willebrand factor (VWF) is critical in facilitating arterial thrombosis. At pathologically high shear rates, the protein unfolds and binds to the arterial wall, enabling the rapid deposition of platelets from the blood. We present a novel continuum model for VWF dynamics in flow based on a modified viscoelastic fluid model that incorporates a single constitutive relation to describe the propensity of VWF to unfold as a function of the scalar shear rate. Using experimental data of VWF unfolding in pure shear flow, we fix the parameters for VWF’s unfolding propensity and the maximum VWF length, so that the protein is half unfolded at a shear rate of approximately $5000\,\text {s}^{-1}$. We then use the theoretical model to predict VWF’s behaviour in two complex flows where experimental data are challenging to obtain: pure elongational flow and stenotic arterial flow. In pure elongational flow, our model predicts that VWF is 50% unfolded at approximately $2000\,\text {s}^{-1}$, matching the established hypothesis that VWF unfolds at lower shear rates in elongational flow than in shear flow. We demonstrate the sensitivity of this elongational flow prediction to the value of maximum VWF length used in the model, which varies significantly across experimental studies, predicting that VWF can unfold between $2000\text { and }3200\,\text {s}^{-1}$ depending on the selected value. Finally, we examine VWF dynamics in a range of idealised arterial stenoses, predicting the relative extension of VWF in elongational flow structures in the centre of the artery compared to high shear regions near the arterial walls.

## Introduction

Coronary heart disease is characterised by the formation of plaque on the walls of arteries leading to the muscles of the heart, restricting blood flow (Casa and Ku 2017). Increased blood flow and pressure from increased physical activity or periods of stress create abnormally high wall shear stress which can lead to the rupture of an existing vulnerable plaque deposit. Following this rupture, a blood clot then rapidly forms to repair the damaged wall (Arroyo and Lee 1999; Bentzon et al. 2014). Blood clot formation in arterial conditions, known as high shear thrombosis, is facilitated by the shear-sensitive blood protein Von Willebrand factor (VWF). This protein has platelet-binding sites along its length and is tightly coiled at normal levels of the fluid shear rate. At pathologically high shear rates, which occur as the blood flow accelerates over the plaque deposit, the protein unfolds and facilitates the formation of a platelet-based clot in the artery.

Von Willebrand factor is a large protein which naturally exists in the blood as a chain of repeating units known as dimers. VWF can be composed of between two and eighty dimers, and the proteins with the most dimers play the most dominant role in haemodynamics (Von 1996; Sadler 1998). VWF shape and length in flow was first revealed in 1996 when atomic force microscopy was used to demonstrate that the protein only unfolds at shear stresses greater than 35 dyne/cm^2^, which is equivalent to a shear rate of $3500\,\text {s}^{-1}$ assuming that the suspending fluid has the viscosity of water (Siediecki et al. 1996). VWF unfolding has since been characterised experimentally for a range of shear rates (Bergal et al. 2022; Lippok et al. 2016; Schneider et al. 2007). All of these works find that VWF unfolds in shear flow with shear rates exceeding approximately $5000\,\text {s}^{-1}$. However, there is significant variation in the maximum length of the protein at high shear rates obtained in the two studies which directly measure VWF extension: Schneider et al. (2007) found that VWF obtains a maximum extension of 15 μm in contrast with the value of 0.17 μm found by Bergal et al. (2022). This variation was attributed in Bergal et al. (2022) to the blurring of VWF in the images obtained at high-flow speeds in Schneider et al. (2007). The experimental works of Schneider et al. (2007) and Bergal et al. (2022) were accompanied by *discrete*, coarse-grained numerical simulations of VWF, in which the groups use contrasting modelling assumptions. Specifically, Schneider et al. (2007) used a collapsed polymer model which best fits the sudden extreme unfolding found in their data whereas Bergal et al. (2022) used an uncollapsed, random chain which predicts more gradual unfolding in line with their experimental results. The work of Lippok et al. (2016) similarly features both experimental and discrete numerical results; however, the authors did not directly image VWF. Instead, the authors subjected VWF to shear force in combination with an enzyme which cleaves the multimers of VWF (ADAMTS13) and assumed that the proteins cleave at a rate proportional to their length. The authors then used a discrete numerical model, assuming that VWF behaves as a collapsed polymer, to relate the cleavage rate of their experiments to the length of VWF. The disparity in the experimental measurements of VWF’s unfolding mechanics adds uncertainty to the design of theoretical models, namely in whether they should predict sudden or gradual unfolding. This is compounded by the further disparity in experimental measurement of VWF length. Together these uncertainties pose a challenge in determining model parameters and therefore to the accuracy of model predictions.

The flow within diseased arteries has complex combinations of regions of shear flow, as well as flow constriction and expansion around narrow regions known as stenoses. However, the small size of VWF combined with the extremely high-flow speeds required to unfold the protein present significant experimental challenges. The limited experimental studies of VWF have all studied its behaviour in simpler setups for instance using pure shear flow, which does not reflect the complexities of physiological arterial flow (see Sect. 1.1). Fu et al. (2017) avoided the problem of tracking the protein at high speed by tethering VWF to a wall. This study yielded extensive quantitative data on the VWF mechanics; however, it also demonstrated that VWF behaviour differs when the protein is tethered to a wall compared to when it is free to move. Specifically, Fu et al. (2017) found that VWF unfolds at low shear rates when tethered, which is likely due to the fact that the protein cannot resist extension by rotating when it is tethered to a wall. As a result of these experimental challenges, the behaviour of VWF in the complex flows that occur within arteries is not well understood. Predicting VWF’s dynamics within arteries is critical to understanding, and ultimately treating, thrombosis in clinically relevant scenarios.

In this paper, we present a novel theoretical model which describes VWF dynamics, we explore VWF dynamics using this model in steady simple two-dimensional flows and in steady, stenotic, arterial flow for Reynolds numbers up to 500, where the Reynolds number is based on the maximum vessel radius. This captures the range of flow rates seen in small arteries near the heart (Mahalingam et al. 2016). In this range, steady flow will remain laminar, since the threshold for turbulence is on the order of Re = 1000 for a stenosed pipe which is 50% obstructed (referred to as a 50% stenosis) (Ahmed and Giddens 1983; Mahalingam et al. 2016). Turbulent flow has been shown to cause cleavage of VWF multimers (Jhun et al. 2023). The model in this paper does not consider the breakage of VWF multimers. Consideration of turbulent flow regimes would require an extension to the model in which proteins are categorised according to their number of monomers, and the transport of these sub-populations in suspension is then tracked.

In Sect. 1.1, we now summarise the characteristics of flow within stenosed arteries. Then, in Sect. 1.2, we review the known behaviour of VWF in response to fluid flows.

### Flow structure within diseased arteries

The action of a fluid flow $\varvec{u}$ on suspended proteins can be described locally by the velocity gradient $\nabla \varvec{u}$. The velocity gradient can be split into symmetric and antisymmetric components:

1 $$\begin{aligned} \nabla \varvec{u}=\frac{1}{2}\left( \nabla \varvec{u}+ \nabla \varvec{u}^T\right) +\frac{1}{2}\left( \nabla \varvec{u}- \nabla \varvec{u}^T\right) =\varvec{D}+\varvec{W}, \end{aligned}$$

where $\varvec{D}$ and $\varvec{W}$ are the rate of strain tensor and the rotation tensor and describe local extension and local rotation, respectively. The magnitude of strain and rotation can be quantified through the scalar shear rate (also referred to as the strain rate), and scalar rotation rate defined as follows:

2 $$\begin{aligned} \dot{\gamma }=\sqrt{2\varvec{D}:\varvec{D}}, \quad \dot{\omega }=\sqrt{2\varvec{W}:\varvec{W}}, \end{aligned}$$

where : denotes the double dot product.

**Fig. 1:**
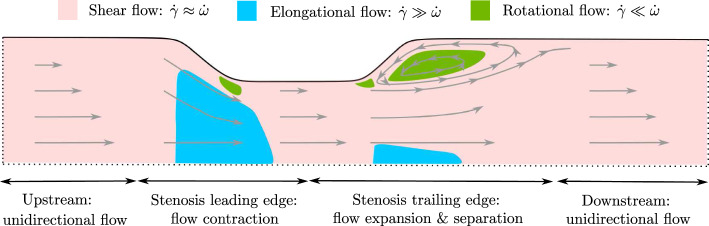
Sketch of the flow structures within a two-dimensional slice of a 3D axisymmetric stenosed artery based on our numerical simulations at $Re=500$ with a 50% stenosis. Dotted lines illustrate the inlet (left), outlet (right), and the centre of the pipe (bottom). Four regions of key flow behaviour are labelled below. Illustrative streamlines are shown by grey arrows. Three key flow structures are highlighted by colours at their respective locations within the artery. Shear flow (pink), which is an approximately equal combination of rotational and elongational flows, occurs near the vessel walls and away from the stenosis where flow is unidirectional. Elongational flow (blue) occurs away from the wall at the leading edge of the stenosis, and to a lesser extent at the trailing edge. Rotational flow (green) occurs predominantly in the recirculation zone behind the stenosis, although small regions of rotation occur close to the vessel wall at the leading and trailing edge of the stenosis

Stenotic arterial flow is a complex combination of elongational and rotational flows (Casa and Ku 2017; Rana et al. 2019). Upstream of the stenosis, the flow is predominantly in the axial direction (assuming that the vessel is not significantly curved) and is categorised as shear flow in which $\dot{\gamma }\approx \dot{\omega }$. The flow accelerates as it reaches a constriction in the vessel leading to an increase in the fluid velocity and the shear rate. We refer to this region as the leading edge of the stenosis. As the flow contracts, for sufficiently steep stenoses, the radial component of the flow becomes comparable to the axial flow, this leads to a region of elongational flow which is defined by $\dot{\gamma }\gg \dot{\omega }$. Just downstream of the stenosis, a region we refer to as the trailing edge of the stenosis, a closed recirculation zone can form in which axial and radial flows are significant. In this region, the flow is rotational, which is defined by $\dot{\gamma }\ll \dot{\omega }$. On both the trailing and leading edge of the stenosis close to the wall, there are small regions of rotational flow as the flow bends significantly to accommodate the stenosis geometry. Far downstream the flow relaxes back to unidirectional. Figure 1 illustrates the locations of these flow structures, based on our numerical simulations at Re = 500 with a 50% stenosis. Details of the simulation method and fluid flow boundary conditions are listed in Sect. 2.2.

To study these complex flow effects in isolation, theoretical and experimental studies often use idealised simple flows for instance; pure elongational flow, in which $\dot{\omega }=0$; pure rotational flow, in which $\dot{\gamma }=0$; and pure shear flow which has exactly equal parts elongation and rotation so that $\dot{\omega }=\dot{\gamma }$. We use these idealised flows to examine the behaviour of our theoretical model in Sect. 3.1, and we use pure shear flow to parameterise our model compared to experimental studies of VWF in Sect. 2.1.1. We now summarise the known behaviour of VWF in different flow structures.

### VWF behaviour in flow and existing theoretical models

The elongational flow within stenotic arteries has been proposed as a key mechanism in VWF’s ability to rapidly unfold (Casa and Ku 2017; Sing and Alexander-Katz 2010). In experimental studies using pure elongational flow, proteins and polymers similar to VWF fully unfold at lower values of shear rate than in pure shear flow (Babcock et al. 2003; Smith et al. 1999); theoretical models predict this behaviour also occurs for VWF in suspension (Sing and Alexander-Katz 2010). However, all experimental studies of VWF unfolding both in free flow and tethered use shear flow, since tracking and imaging proteins in suspension at high shear rates is less challenging in unidirectional flows (Bergal et al. 2022; Fu et al. 2017; Schneider et al. 2007). No experimental studies to date have examined VWF dynamics in pure elongational flow. As a result, the hypothesis that there is also a lower shear rate threshold of unfolding in pure elongational flow for VWF has not yet been tested *in vitro*. Furthermore, in elongational flow regions where $\dot{\gamma }\gg \dot{\omega }$, it is unclear how much the shear rate must exceed the rotation rate for the proposed rapid unfolding to occur. Babcock et al. (2003) examined DNA molecules in elongational flow and determined that DNA unfolds more easily if the difference between the shear rate and rotation rate divided by the total rotation and shear, $(\dot{\gamma }-\dot{\omega })/(\dot{\gamma }+\dot{\omega })$, exceeds 0.0048. However, this threshold has not yet been characterised for VWF.

Mathematical models can examine VWF’s dynamics in flows that are challenging to generate in vitro, namely elongational flows. Existing mathematical models of VWF are predominantly discrete models which describe the protein as a chain of beads and springs. The spring coefficients can then be parameterised so that the model predicts VWF unfolding at approximately 5000$\,\text {s}^{-1}$ in pure shear flow to match experimental data as measured by Schneider et al. (2007). However, these bead and spring models of VWF predict a wide variety of unfolding thresholds in pure elongational flow: 500$\,\text {s}^{-1}$ (Sing and Alexander-Katz 2010), 2400$\,\text {s}^{-1}$ (Nguyen et al. 2021), 2500$\,\text {s}^{-1}$ (Kania et al. 2021), and 3500$\,\text {s}^{-1}$ (Dong et al. 2019). Discrete models can also examine VWF’s interactions with red blood cells or platelets in flow as part of the thrombosis cascade. For instance, (Rack et al. 2017) demonstrate that the proteins remain globular in the centre of the vessel which enables the protein to travel to the edge of the vessel more easily since collisions with red blood cells displace globular proteins further than the unfolded proteins. Liu et al. (2022) examine the formation of small platelet–VWF aggregates and predict the required protein concentration and length to initiate aggregation. We note that these numerical studies predate the contrasting, gradual VWF unfolding behaviour found by Bergal et al. (2022) and therefore describe VWF using collapsed polymer models which predict sudden unfolding.

Discrete models of VWF can be characterised with in vitro data and offer insights into protein mechanics. However, these models can only accommodate a limited number of proteins and their interactions during thrombosis before their numerical solution becomes demanding. An alternative approach is to employ continuum models that examine the dynamics of a large number of constituents such as platelets, red blood cells and proteins, together with their role in thrombosis (Du et al. 2020; Leiderman and Fogelson 2011; Wu et al. 2020). To explicitly incorporate VWF into these models, a continuum description for VWF able to describe the protein’s dynamics in complex, evolving arterial flow is required.

VWF dynamics are modelled using a continuum framework in Zhussupbekov et al. (2021). The authors use a two-species model where VWF exists in one of the two binary states: either fully unfolded or completely globular. Each species is tracked using an advection–diffusion equation. The unfolding rate which moves proteins from the globular category to the unfolded category is modelled by first classifying the local flow as approximately pure shear, pure elongational, or pure rotational, then prescribing unfolding rates in each case. The unfolding of VWF in shear flow uses the unfolding rate of Lippok et al. (2016) derived from cleavage data. In regions defined as elongational flow, according to the DNA threshold of Babcock et al. (2003), the authors use a constant unfolding rate. No unfolding occurs in rotational flow. This model predicts that, in a stenotic flow, a significant number of VWF molecules are unfolded both close to the stenosis wall in the shear flow region and away from the wall due to elongational flow regions. This model was then incorporated into a thrombosis model where shear-flow-induced VWF unfolding near the wall was shown to match the location of thrombus formation in vitro (Zhussupbekov et al. 2022). This is the first work to include an explicit description of unfolding VWF in a continuum model. Other studies include VWF by increasing the phenomenological binding rate between platelets and the vessel wall as a function of shear rate or elongation rate (Du et al. 2020; Sorensen et al. 1999; Wu et al. 2020).

In this paper, we present a novel continuum model for VWF that predicts the length and orientation of the protein in varying flow structures. Our model describes VWF length and orientation continuously, allowing examination of cases where VWF only partially extends which is vital to examine thrombosis at shear rates marginally outside of the normal range. Our model does not split the local flow into discrete categories. Instead, our model encodes the flow structure through the velocity gradient and can, therefore, describe VWF dynamics in shear, elongational, and rotational flows and combinations of these in three dimensions using a single unfolding propensity. This unfolding propensity function can be parameterised using experimental data from pure shear flow which eliminates the need to use data from other proteins as in Zhussupbekov et al. (2021) which may be inaccurate for VWF. Crucially, this allows us to predict the protein unfolding throughout the full range of flow types that occur within diseased arteries. The accuracy of these predictions relies on the corresponding accuracy of our model parameters. In this paper, we quantify how model predictions change depending on the selected value of the parameter for which the experimental measurements are the most uncertain: the maximum length VWF can reach in flow.

It is important to note which VWF behaviours the model does not currently include. Firstly, VWF has been shown to demonstrate hysteresis, whereby the time taken for the protein to relax back to its natural length following the removal of flow is much longer than the time taken to unfold when the flow is applied. Fu et al. (2017) demonstrated that tethered molecules unfold over approximately 0.01 s when the flow is turned on and require approximately 1 s to return to their natural length. The time required to travel the length of the coronary artery can be estimated to be approximately 1 s (based on an arterial length of 10 cm and a velocity of 0.1 m/s (Grief and Richardson 2005)). However, the time required to pass a typical stenosis is approximately 0.1 s (based on a 1.7 cm stenosis and a 0.16 m/s pathological velocity (Elhfnawy et al. 2019; Zafar et al. 2014). This means that the proteins could remain partially unfolded in the region downstream of the stenosis. In this paper, we do not consider VWF hysteresis, but it is a valuable extension discussed in Sect. 4. Finally, in shear flow with a constant shear rate above 5000$\,\text {s}^{-1}$, the protein periodically unfolds, tumbles, and then refolds. The period between this unfolding and refolding and the magnitude of unfolding both increase as the shear rate increases (Sing and Alexander-Katz 2010). We note that the continuum model we present in this paper to describe VWF’s dynamics does not capture these unfolding and refolding cycles, but rather the average length that an individual protein has in flow.

The paper is structured as follows. First, in Sect. 2.1, we present the mathematical model which is derived from an existing viscoelastic fluid model. In Sect. 2.2, we present an idealised arterial stenosis flow setup. In Sect. 3.1, we explore the predictions for VWF behaviour in pure shear flow and pure elongational flow, we do not examine pure rotational flow as VWF does not extend in this regime. In Sect. 3.2, we determine the sensitivity of this elongational flow prediction to the value of maximum VWF length used in the model, which varies significantly between the experiments of Schneider et al. (2007) and Bergal et al. (2022). In Sect. 3.3, we explore the mechanistic insight that our model can provide in the complex flow regimes inside arteries through numerical simulations in a range of idealised stenoses. The flow consists of predominantly shear flow near the stenosis wall and regions of predominantly elongational flow at the leading edge of the stenosis. We select the maximum VWF extension as found in Schneider et al. (2007) and show that the model can predict the relative extension of VWF in the elongational flow structures in the centre of the artery compared to high shear regions near the arterial walls. For this value of maximum VWF extension, we find that VWF is most extended and, therefore, most reactive with platelets, in the shear flow close to the stenosis wall. We conclude in Sect. 4 by discussing the implications of these predictions, how they can be used to examine VWF’s role in arterial thrombosis and highlighting the limitations of the model.

## Methods

### VWF model

We model blood, which contains VWF, using the Navier–Stokes equations and a modified Finitely Extensible Nonlinear Elastic model with the Peterlin spring closure (FENE-P) in the limit where the contribution to the fluid stress from the suspended VWF molecules is negligible. The relative scale of the protein stress compared to the stress of the suspending fluid is determined by the ratio $Gd/\mu U$, where *d* is a reference lengthscale, *U* is a reference velocity value, and $\mu$ is the fluid viscosity and $G=nk_bT$, in which *n* is the number of proteins per meter cubed, $k_\mathrm{{b}}$ is Boltzmann’s constant, and *T* is the average temperature. To estimate the number of proteins per meter cubed, we use the concentration of VWF in the blood (0.055 g/m^3^) and the protein’s molecular weight (between 500 and 20,000 KDa depending on the number of dimers combined) (Von 1996; Peyvandi et al. 2011). The value of *G* can then be estimated as between 0.027 and 6.7$\times 10^{-4}$ Pa. In this paper, we consider arterial flows with fluid velocities between 0.17 and 0.84 m/s in a 1.5 mm radius vessel. Hence, the maximum value of the ratio $Gd/\mu U$ is approximately 0.008, demonstrating that VWF’s contribution to the fluid stress is minimal. As a result, the flow is uncoupled from the VWF dynamics, and VWF does not contribute to the overall fluid stress.

We model blood as an incompressible, Newtonian, viscous fluid with velocity $\varvec{u}$ and pressure *p* at time *t*. The flow is governed by the incompressible Navier–Stokes equations given by

3 $$\begin{aligned} \nabla \cdot \varvec{u}=0, \quad \rho \left( \frac{\partial \varvec{u}}{\partial t}+ \varvec{u}\cdot \nabla \varvec{u}\right) =- \nabla p+\mu \nabla ^2\varvec{u}, \end{aligned}$$

where the density $\rho$ and viscosity $\mu$ of the blood are taken to be constant.

We capture the average length and orientation of VWF molecules via the symmetric, rank 2, configuration tensor $\varvec{A}.$ This tensor description of a protein in flow can be rigorously derived from dynamics of a two-bead dumbbell connected by a nonlinear spring (Bird et al. 1980). The tensor’s components, $A_{ij}$, represent the average of the components of the end-to-end vector of the microscopic dumbbell $\varvec{r}$ such that $A_{ij}=<r_ir_j>$. Hence, the diagonal components of $\varvec{A}$ represent the average length in each axis direction squared, e.g. $A_{xx}=<r_x^2>$ for the $x-$ direction. Hence, the components of $\varvec{A}$ can be used to describe the protein’s orientation and extension in each direction, which we will demonstrate for simple flows in Sect. 3.1.

The trace of $\varvec{A}$ is proportional to the average length of the protein squared (Rallison and Hinch 1988), and hence, we define the normalised VWF length as

4 $$\begin{aligned} \mathcal {L}=\sqrt{\frac{Tr(\varvec{A})}{Tr(\varvec{I})}}, \end{aligned}$$

so that when $\mathcal {L}=1$, the protein is at its natural length for which $\varvec{A}=\varvec{I}$. We define the extension of the proteins to be $\mathcal {E}=\mathcal {L}-1$, which we use to compare model predictions to experiments in Sect. 2.1.1.

The configuration tensor evolves as a FENE-P fluid and is governed by the following equation:

5 $$\begin{aligned} \frac{\partial \varvec{A}}{\partial t}+ \varvec{u}\cdot \nabla \varvec{A}-\varvec{A}\cdot \nabla \varvec{u}-&( \nabla \varvec{u})^T\cdot \varvec{A}=-\frac{1}{\tau (\dot{\gamma })}\left( \varvec{f}(\varvec{A})\varvec{A}-a\varvec{I}\right) , \end{aligned}$$

where ${\tau }$ is the VWF relaxation time, $f(\varvec{A})=\text {L}^2/(\text {L}^2-Tr(\varvec{A}))$ is the nonlinear spring law which restricts the protein length to be less than a prescribed maximum we denote L, and $a=\text {L}^2/(\text {L}^2-Tr(\varvec{I}))$ is a constant which ensures that in the absence of flow $\varvec{A}=\varvec{I}$ (Bird et al. 1980). In Eq. (5), the first two terms on the left-hand side describe the transport of proteins along fluid streamlines. The last two terms on the left-hand side represent the effects of the rotation and extension from the fluid flow which arise since the two ends of the protein can experience different fluid velocities. The right-hand side is a nonlinear function of the configuration tensor which represents the elastic forces which resist extension and also maintain the protein’s finite length.

We model the unfolding of VWF at high shear rates by allowing the VWF relaxation time ${\tau }$ in (5) to depend on the shear rate $\dot{\gamma }$. This is described through a saturating function of the fluid shear rate as follows:

6 $$\begin{aligned} \tau (\dot{\gamma })=\alpha \left( \frac{1}{2}(\tanh (\beta (\dot{\gamma }-\gamma ^*))+1)+\delta \right). \end{aligned}$$

**Fig. 2:**
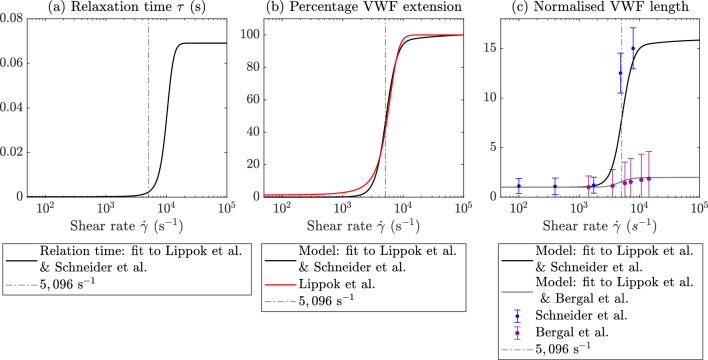
**a** Modified relaxation time of VWF, Eq. (6). At low shear rates, the relaxation time is small, and the proteins do not extend; at high shear rates, the relaxation time is larger which allows the proteins to extend, this is fitted using the unfolding curve of Lippok et al. (2016). **b** Extension of VWF in pure shear flow for varying shear rate compared to the normalised VWF extension curve of Lippok et al. (2016) (red); extension is normalised so that the maximum value is one. **c** VWF length $\mathcal {L}$ for varying shear rate in pure shear flow compared to the experimental data of (Schneider et al. 2007) (blue) and (Bergal et al. 2022) (purple), both of which are normalised by the minimum length measured. When using L $=22.6$ to match Schneider et al. (2007), VWF reaches 50% of its maximum length at 5096$\,\text {s}^{-1}$ (dashed-dot line). The grey curve shows model fitting using L$=2.8$ to match Bergal et al. (2022), this provides a quantitative match however predicts more sudden unfolding compared to Bergal et al. (2022). All subfigures use parameters listed in Table 1 fitted with L$\,=22.6$, aside from the grey curve in (**c**) which uses L $=2.8$

The parameter $\gamma ^*$ is the shear rate at which VWF relaxation time is half of its maximum value, and $\beta$ describes how quickly the relaxation time varies as the shear rate increases. Large values of $\beta$ correspond to a rapid increase in $\tau$ once the shear rate reaches $\gamma ^*$. Finally, the parameters $\alpha$ and $\delta$ fix the minimum and maximum values of the relaxation time to be $\alpha \delta$ and $\alpha (1+\delta )$, respectively. This nonlinear relaxation time is shown in Fig. 2a. Examining the left- and right-hand sides of (5), VWF extension is driven by fluid gradients, which are proportional to the shear rate $\dot{\gamma }$, and extension is resisted by elastic forces, which are proportional to the inverse relaxation time, $1/\tau$. The relative size of these two effects is determined by $\dot{\gamma }\tau$, so that if $\dot{\gamma }\tau \ll 1$, then elastic forces dominate and the protein remains globular whereas if $\dot{\gamma }\tau \gg 1$, fluid extension forces dominate and the protein unfolds. In practice, this means that we select the values of the unfolding parameters $\alpha$ and $\delta$, so that $\alpha (1+\delta )\dot{\gamma }\gg 1$ for shear rates, where we want VWF to unfold and $\alpha \delta \dot{\gamma }\ll 1$ at shear rates where VWF remains globular. We detail the parameter selection method in Sect. 2.1.1.

There are several important points to note when applying this model. Firstly, the FENE-P model describes dilute suspensions and does not include protein–protein interactions. This means that we cannot model entanglement or protein–protein binding which may be significant in the later stages of thrombosis. Secondly, the FENE-P equation is derived through mean-field analysis of a collection of microscopic Brownian dumbbells in the absence of walls (Bird et al. 1980). However, despite this inconsistency, the model is extensively and successfully used for flows in bounded domains. Including boundary effects in viscoelastic models remains an open theoretical challenge; hence, in this paper, we use the FENE-P model to describe the dynamics of VWF in bounded flows. We use the model solution at the boundary to describe the length of VWF close to the wall; gaining insight into the protein dynamics where VWF–platelet binding occurs. Finally, we note that our modified FENE-P model cannot predict VWF hysteresis, since (5) is single-valued for a particular shear rate. Hence, the proteins will relax back to their natural length on the same timescale as they unfolded on. We discuss the limitations of these assumptions on model predictions in Sect. 4.

#### Parameterisation

The model parameters required to describe the flow and VWF behaviour according to (3) and (5) are shown in Table 1. The VWF unfolding parameters in (6), namely $\alpha$, $\gamma ^*$, $\beta$, $\delta$, and the maximum VWF length L, are unknown.

We estimate these parameters, aside from L, by fitting the numerical solution of the FENE-P equation in simple shear flow to the normalised VWF extension curve obtained by Lippok et al. (2016). We use a gradient-based minimiser in *Matlab* to carry out the fitting. The curve of (Lippok et al. 2016) provides relative VWF extension across a large range of shear rates and shows the protein reaching a maximum extension. However, since the authors measured VWF cleavage then fitted a discrete numerical model to obtain this unfolding curve, this study does not provide the maximum VWF length L. The value of extension VWF achieves in flow is not well established experimentally; measured VWF maximum length ranges from twice its natural length to fifteen times its natural length (Bergal et al. 2022; Schneider et al. 2007). To quantify how our predictions for VWF behaviour in pure elongational flow would change as more data becomes available on VWF length, in Sect. 3.2, we estimate model parameters, $\alpha$, $\gamma ^*$, $\beta$, $\delta$, using the normalised VWF extension curve obtained by Lippok et al. (2016) for a range of L values from 2.8 to 22.6. This corresponds to a maximum length between twice (as found by Bergal et al. 2022) and fifteen times the protein’s natural length (as found by Schneider et al. (2007)). Details of the parameter estimation algorithm are given in Appendix A.

For simplicity, in Sects. 3.1 and 3.3, we present VWF dynamics for a fixed value of L, namely L$\,=22.6$, so that the maximum possible extension of VWF matches the value of 15 $\mu$m obtained by Schneider et al. (2007) when normalised by the globular length of 1 $\mu$m. The normalised VWF extension in pure shear flow for L$\,=22.6$ is compared to the normalised VWF extension obtained by Lippok et al. (2016) in Fig. 2b. The corresponding VWF length is also compared with the data of (Schneider et al. 2007) in Fig. 2c. Our fitted model finds that VWF reaches 50% of its maximum length at 5096$\,\text {s}^{-1}$ in pure shear flow, this is within 1% of the unfolding threshold found by Lippok et al. (2016) of 5122$\,\text {s}^{-1}$. Finally, in Fig. 2c, we also show our model fitting which uses L $=2.8$ to match the maximum length measured in flow from Bergal et al. (2022). This case of our model obtains a quantitative match to the minimum and maximum extent of VWF unfolding found by Bergal et al. (2022); however, we predict that VWF will unfold more suddenly, this is as expected as we have used the unfolding curve from Lippok et al. (2016) to determine all parameters aside from *L*.

**Table 1:** Dimensional model parameters. The VWF parameters, aside from L, have been estimated by fitting VWF extension in shear flow to the unfolding curve from (Lippok et al. 2016). In Sects. 3.1 and 3.3, the maximum VWF length is fixed at L$\,=22.6$ to match data of (Schneider et al. 2007). In Sect. 3.2, L is varied

Name	Param	Value	Units	Source
Viscosity of blood	$\mu$	0.0025	Pa s	(Pries et al. 1992)
Density of blood	$\rho$	1050	kg m^-3^	(Vitello et al. 2015)
Extension parameter	$\alpha$	0.069	s	Fit to (Lippok et al. 2016)
Extension parameter	$\beta$	$3.44\times 10^{-4}$	s	Fit to (Lippok et al. 2016)
Extension parameter	$\delta$	$9.70\times 10^{-4}$	–	Fit to (Lippok et al. 2016)
Extension parameter	$\gamma ^*$	$1.0\times 10^{4}$	$\text {s}^{-1}$	Fit to (Lippok et al. 2016)
Maximum VWF length	L	S.3.3 & 3.1: 22.6,	–	(Schneider et al. 2007)
		S.3.2: $2.8-22.6$		–

### Arterial flow setup

**Fig. 3:**
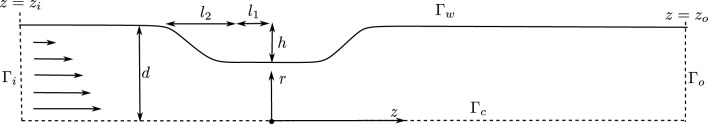
Diagram of (*r*, *z*)-plane of our axisymmetric arterial-scale stenosis geometry. Cylindrical polar coordinates (*r*, *z*) are marked. The stenosis is symmetric around $z=0$ and is defined by parameters $l_1$, $l_2$, and *h* which define the length, steepness, and height. The inlet at $z=z_\mathrm{{i}}$ is $\Gamma _i$, the outlet at $z=z_o$ is $\Gamma _o$, the walls are $\Gamma _\mathrm{{w}}$, and the pipe centre line is marked $\Gamma _\mathrm{{c}}$

We examine our model predictions of VWF unfolding in an idealised axisymmetric stenosis under steady flow for a range of flow speeds and geometries. The arterial stenosis geometry is shown in Fig. 3, the stenosis is defined by its height *h*, half-length $l_1$, and steepness $h/l_2$. The pipe radius *d* is chosen to match the dimensions of the coronary artery. We solve the model in the (*r*, *z*)-plane assuming axisymmetry, as illustrated in Fig. 3. In the (*r*, *z*)-plane, the inlet of the pipe is located at $z=z_i$ and is denoted $\Gamma _{i}$, the outlet is located at $z=z_o$ and is denoted $\Gamma _{o}$, the pipe walls which include the stenosis are denoted $\Gamma _{w}$, and the centre of the pipe at $r=0$ is denoted $\Gamma _{\mathrm{{c}}}$. We denote the fluid flow components as *w* and *u* in the axial and radial directions, respectively.

To close our model in this geometry, we prescribe boundary conditions for (3) and (5) as follows. The flow is driven by a unidirectional, parabolic inlet flow on $\Gamma _\mathrm{{i}}$ with maximum velocity *U*. At the outlet, $\Gamma _\mathrm{{o}}$, we prescribe that the normal stress is equal to a prescribed pressure, $p_a$, and that the flow is unidirectional. The latter condition creates a requirement for the pipe to be longer than any recirculation zone behind the stenosis. We prescribe no slip on the walls of the domain $\Gamma _\mathrm{{w}}$. On the centre of the domain, $\Gamma _\mathrm{{c}}$, we prescribe no normal flow and a symmetry condition that the normal derivative of the axial flow vanishes. At the inlet, $\Gamma _\mathrm{{i}}$, we prescribe an inlet configuration of VWF, $\varvec{A}_{\text {in}}(r)$, which is the solution of Eq. (5) under the imposed inlet parabolic flow. Since we consider steady flow, we analyse the steady counterparts of (3) and (5) and do not require initial conditions to close the problem.

#### Dimensionless model

The dimensionless model is obtained by scaling lengths with the maximum pipe radius and the fluid velocity components with the maximum inlet velocity. Hence, we define dimensionless variables, denoted with hats, as follows:

7 $$\begin{aligned} z&= \mathrm{{d}}\hat{z},&\quad r&= \mathrm{{d}}\hat{r},&\quad p&=p_a+ \rho U^2 \hat{p},&\quad \varvec{u}&=U\hat{\varvec{u}}, \end{aligned}$$

where we note that the configuration tensor is dimensionless so does not need rescaling. The pressure scaling in (7) is defined to balance inertial forces and the pressure gradient in (3) relative to the prescribed outlet pressure $p_a$. The dimensionless shear rate is defined as $\dot{\gamma }=(U/d)\hat{\dot{\gamma }}$. Using (7) the stenosis geometry is defined by its height $\hat{h}=h/d$ and the lengths $\hat{l}_1=l_1/d$ and $\hat{l}_2=l_2/d$. Inserting scalings (7) into (3) and (5) and dropping hats on dimensionless variables, we recover the dimensionless steady Navier–Stokes and FENE-P equations given by

8 $$\begin{aligned} \nabla \cdot \varvec{u}=0, \quad \mathrm{{Re}}\,\varvec{u}\cdot \nabla \varvec{u}&=-\mathrm{{Re}} \nabla p+ \nabla ^2 \varvec{u}, \end{aligned}$$

9 $$\begin{aligned} \xi \mathrm{{Re}} ( \varvec{u}\cdot \nabla \varvec{A}-\varvec{A}\cdot \nabla \varvec{u}- \nabla \varvec{u}^T\cdot \varvec{A})&=-\frac{1}{\hat{\tau }(\dot{\gamma })}(f(\varvec{A})\varvec{A}-a\varvec{I}), \end{aligned}$$

where the Reynolds number is $\mathrm{{Re}}=\rho U\mathrm{{d}}/\mu$, and $\xi =\alpha \mu /\mathrm{{d}}^2\rho$ is defined so that the product $\xi Re$ is the Deborah number, which represents the ratio of the timescales of protein relaxation to fluid advection. However, we choose to work with $\xi$ rather than the Deborah number so that we are able to examine the system for varying Re. The FENE-P function $f(\varvec{A})=\text {L}^2/(\text {L}^2-Tr(\varvec{A}))$ and $a=\text {L}^2/(\text {L}^2-Tr(\varvec{I}))$ remain unchanged as L is dimensionless. The dimensionless VWF relaxation time is

10 $$\begin{aligned} \hat{\tau }(\dot{\gamma })=\frac{1}{2}\left( \tanh {\left( \hat{\beta }\mathrm{{Re}}\left( \dot{\gamma }-\frac{\hat{\gamma }^*}{\mathrm{{Re}}}\right) \right) }+1\right) +\delta , \end{aligned}$$

where $\hat{\beta }=\beta \mu /\mathrm{{d}}^2\rho$ and $\hat{\gamma }^*=\gamma ^*\rho \mathrm{{d}}^2/\mu$ are the dimensionless relaxation time parameters. The dimensionless boundary conditions for the system are

11 $$\begin{aligned} w=\left( 1-r^2\right) , \quad u=0, \quad \varvec{A}=\varvec{A}_{\text {in}}(r)\quad&\text { on } \quad&\Gamma _{i}, \end{aligned}$$

12 $$\begin{aligned} \hat{\varvec{n}}\cdot \varvec{\sigma } \cdot \hat{\varvec{n}}=0, \quad u=0 \quad&\text { on } \quad&\Gamma _{o},\end{aligned}$$

13 $$\begin{aligned} u=0, \quad \frac{\partial w}{\partial r}=0 \quad&\text { on } \quad&\Gamma _{c}, \end{aligned}$$

14 $$\begin{aligned} \varvec{u}=\varvec{0} \quad&\text { on } \quad&\Gamma _{w}. \end{aligned}$$

In (11), the inlet configuration of VWF, $\varvec{A}_{\text {in}}(r)$, is the solution of the dimensionless Eq. (9) under the imposed inlet parabolic flow. Dimensionless parameters and the values used in our numerical simulations in Sects. 3.1 and 3.3 are shown in Table 2. In Sect. 3.2, we vary the VWF unfolding parameters, namely $\alpha$, $\gamma ^*$, $\beta$, $\delta$, and L. For the arterial flow simulations in Sect. 3.3, we place the channel outlet at $\hat{z}_o=30+\hat{l}_1+\hat{l}_2$ which is sufficient to ensure the domain extends beyond the fluid recirculation zone for $\hat{h}=0.5$.

**Table 2:** Dimensionless model parameters used in Sects. 3.1 and 3.3, those with ranges are varied, all others held fixed

Name	Param.	Definition	Value(s)
Dimensionless pipe outlet	$\hat{z}_o$	$z_o/d$	$30+\hat{l}_1+\hat{l}_2$
Dimensionless pipe inlet	$\hat{z}_i$	$z_i/d$	-10
Dimensionless stenosis height	$\hat{h}$	*h*/*d*	0.3–0.5
Dimensionless stenosis length	$\hat{l}_1$	$l_1/d$	1.5
Dimensionless stenosis parameter	$\hat{l}_2$	$l_2/d$	$2-5$
Reynolds number	Re	$\rho U \mathrm{{d}}/\mu$	$200-500$
VWF extension parameter	$\hat{\beta }$	$\beta \mu /\mathrm{{d}}^2\rho$	$2.16\times 10^{-4}$
VWF extension parameter	$\delta$	–	$9.7\times 10^{-4}$
VWF extension parameter	$\xi$	$\alpha \mu /\mathrm{{d}}^2\rho$	0.043
VWF extension parameter	$\hat{\gamma }^*$	$\gamma ^*\mathrm{{d}}\mathrm{{d}}^2\rho /\mu$	$1.60\times 10^4$
Maximum VWF length	L	–	22.6

#### Numerical method

For an illustrative range of stenosis geometries, we consider a range of Reynolds numbers from 200 to 500 which produce shear rates representative of diseased arteries (Casa and Ku 2017). We solve the model using the Finite Element Method implemented using the Python Package FEniCS (Logg and Wells 2010; Logg et al. 2012) which allows implementation of the weak form in the language UFL (Logg et al. 2012; Kirby and Logg 2006; Ølgaard and Wells 2010). This problem is then compiled by FIAT (Kirby 2004, 2012). We use GMSH to construct a mesh of the stenosis geometry (Geuzaine and Remacle 2009). Since the flow is independent of the VWF configuration, we first solve for the fluid flow and then the VWF dynamics. Full details of the numerical method are given in Appendix B.

We add artificial diffusion to the FENE-P equation with a Péclet number of $10^{3}$ following the regularisation procedure commonly applied during the numerical solution of viscoelastic fluid models at high Reynolds numbers (Guy and Thomases 2014; Sureshkumar and Beris 1995). Artificial diffusion allows the hyperbolic equation for the VWF configuration tensor to be solved using the finite element method and avoids instability at locations where the shear stress changes rapidly. The inclusion of artificial diffusion means that we must prescribe boundary conditions for the configuration tensor on all boundaries. We prescribe a symmetry condition, $\nabla \varvec{A}\cdot \hat{\varvec{r}}=\varvec{0}$, on the centre of the pipe. On solid walls, there are two approaches commonly used in existing numerical studies; firstly, Dirichlet boundary conditions can be applied where the tensor $\varvec{A}$ is set to equal the solution of (9) in the absence of flow as in Paulo et al. (2014); Sureshkumar and Beris (1995). Secondly, no normal diffusive flux can be applied on the walls as in Richter et al. (2010). We adopt the latter approach as it reduces computational complexity: No diffusive flux boundary conditions can be easily applied during the Finite Element Method solution, and Dirichlet conditions would require the additional solution of the FENE-P model on the walls by an alternative method. We note that since the FENE-P equation was derived in the absence of walls, the choice of boundary conditions when artificial diffusion is added is an open question for both the FENE-P model and other viscoelastic fluid models (El-Kareh and Leal 1989).

## Results

We now demonstrate how our model can be used to gain insight into VWF’s behaviour in experimental flows and make predictions of the protein’s dynamics in complex flow regimes. We first consider the simpler flow regimes of pure shear flow and pure elongational flow in Sects. 3.1 and 3.2, and then, we consider stenotic arterial flow in Sect. 3.3.

### VWF behaviour in pure shear and elongation flow

In this section, we examine steady, spatially independent solutions of our VWF model in two-dimensional pure shear flow and two-dimensional pure elongational flow. To quantitatively compare the solutions, we set the flows to have the same scalar shear rate, $\dot{\gamma }$. We use a Cartesian coordinate system (*x*, *y*) with corresponding basis vectors $(\varvec{i},\varvec{j})$. We note that in two-dimensional pure rotational flow with $\varvec{u}=\dot{\omega }(y\varvec{i}-x\varvec{j})$, the solution of (5) is $\varvec{A}=\varvec{I}$, which implies that the proteins remain at their natural length, and are randomly oriented. Hence, as expected, rotational flow only rotates the proteins but does not extend them.

We take the velocity field of the pure shear flow to be $\varvec{u}=\dot{\gamma }y\varvec{i}$, where $\dot{\gamma }$ is the shear rate. In two dimensions, the configuration tensor has three unique components as a result of symmetry, where $A_{xx}$ and $A_{yy}$ are the average lengths squared in *x*- and *y*-directions, respectively. We seek a configuration tensor independent of time and space, which is possible since the shear rate is spatially uniform. In this case, we find that Eq. (5) reduces to an algebraic system:

15 $$\begin{aligned} 2\dot{\gamma }{\tau }A_{xy}=f(\varvec{A})A_{xx}-a,\ \dot{\gamma }{\tau }A_{yy}=f(\varvec{A})A_{xy},\ f(\varvec{A})A_{yy}=a. \end{aligned}$$

We take the velocity field of pure elongational flow to be $\varvec{u}=\dot{\gamma }\left( x\varvec{i} -y\varvec{j}\right) /{2}$, where again $\dot{\gamma }$ is the shear rate (for pure elongational flow, $\dot{\gamma }$ is sometimes referred to as the elongation rate). As in pure shear, we seek a steady, spatially independent solution of (5) which gives the following algebraic system:

16 $$\begin{aligned} -2\dot{\gamma }{\tau }A_{xx}=f(\varvec{A})A_{xx}-a,\ 2\dot{\gamma }{\tau }A_{yy}=f(\varvec{A})A_{yy}-a,\ A_{xy}=0, \end{aligned}$$

so that the configuration tensor is diagonal, reflecting that the directions of principal stretch are the *x*- and *y*-axes.

**Fig. 4:**
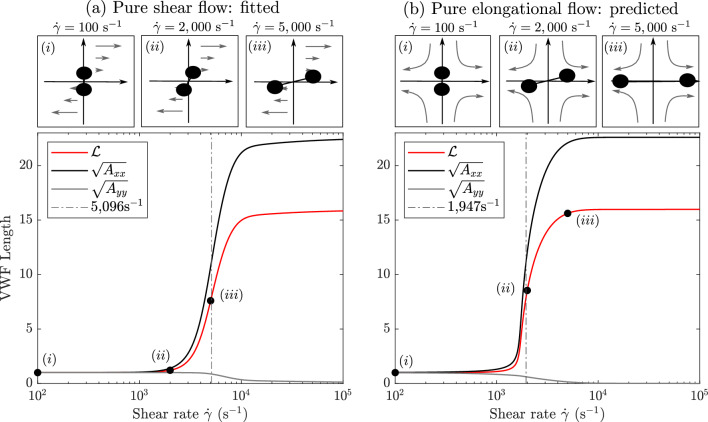
Numerical solutions of the VWF model are shown with insets (*i*)–(*iii*) above of typical VWF’s length and alignment at three increasing shear rates. **a** In pure shear flow, the protein first extends in the *x*-direction, then contracts in the *y*-direction to maintain finite length. The dashed line shows 5096 s^-1^ at which VWF is half unfolded. **b** In pure elongation flow, the protein extends in the *x*-direction and contracts in the *y*-direction simultaneously resulting in full unfolding at lower shear rates than in shear flow. The dashed line shows 1947 s^-1^ at which VWF is half unfolded. VWF parameters listed in Table 2 with L$\,=22.6$

The numerical solutions of (15) and (16) for increasing shear rate are shown in Fig. 4a, b respectively. For each flow type, illustrations of VWF behaviour at three increasing shear rates are shown. Considering first the solution in pure shear flow, Fig. 4a, we see that for values of the shear rate below the unfolding threshold, we have $\varvec{A}\approx \varvec{I}$; this represents a globular protein as shown in inset (*i*). At $\dot{\gamma }\approx 2000$ s^-1^, the protein is only slightly unfolded, as shown in inset (*ii*). At large shear rates, $\dot{\gamma }\approx 5000$ s^-1^, the protein is 50% unfolded and begins to align in the *x*-direction, as shown in inset (*iii*). As the shear rate increases further, to maintain the finite-length restriction enforced by the VWF model, the protein’s length in the *y*-direction tends to zero. We have fitted our model behaviour in shear flow to the unfolding curve of (Lippok et al. 2016) to obtain that at $\dot{\gamma }=5096$ s^-1^, the protein is unfolded to half its maximum length, which is within 1% of the value obtained by Lippok et al. (2016) of 5122 s^-1^. The 50% unfolding threshold is shown by the dot-dash vertical line in Fig. 4a.

In elongational flow, shown Fig. 4b, VWF remains globular for $\dot{\gamma }<100$ s^-1^, as shown in inset (*i*). However, at $\dot{\gamma }\approx 2000$ s^-1^, the protein is 50% unfolded in the *x*-direction and contracted in the *y*-direction, as illustrated in inset (*ii*). This is in contrast with pure shear flow, where contraction in the *y*-direction only occurs at larger shear rates to maintain the protein’s finite length. We predict that in elongational flow, when using L$\,=22.6$, the proteins will be 50% unfolded at $\dot{\gamma }=1947$ s^-1^, which is marked on Fig. 4b by the dash-dotted line. Since pure shear flow is the superposition of a pure elongational flow and a pure rotational flow, VWF extends to its maximum length at a much lower shear rate in pure elongational flow as rotation allows the protein to avoid unfolding. This demonstrates that our model reflects this well-established property of polymers and proteins in flow which is predicted to also occur for VWF (Bird et al. 1980; Sing and Alexander-Katz 2010). For both pure elongational flow and pure shear flow, our modified relaxation time ensures that the proteins remain globular at low shear rates, further reflecting known VWF behaviour (Casa and Ku 2017).

**Fig. 5:**
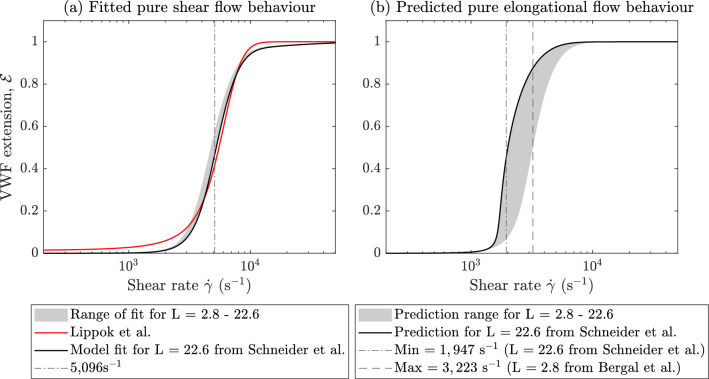
**a** VWF model in pure shear flow compared to (Lippok et al. 2016), the range of fitted curves obtained as L varies between 2.8 and 22.6 is shown in grey. For all values of L, we are able to obtain a close quantitative match to the Lippok et al. (2016) curve. **b** The corresponding range in predicted VWF behaviour in pure elongational flow is shown in grey. The dot-dashed line shows the minimum 50% unfolding threshold, found with L$\,=22.6$, and the dashed line shows the maximum unfolding threshold which is found for L$\,=2.6$. In both plots, black lines show the model solutions with L$\,=22.6$

### Pure elongational flow predictions: parameter sensitivity

In Sect. 3.1, the VWF parameters, listed in Table 2, were fitted to the unfolding curve of (Lippok et al. 2016) with the maximum VWF length, L, fixed at 22.6. Using this value of L, we predicted that VWF will be 50% unfolded at $\dot{\gamma }=1947$ s^-1^ in pure elongational flow. Since the extent of VWF unfolding in vitro is not well established, in this section, we vary the maximum VWF length to determine the range of pure elongational unfolding rates which can be predicted by our model.

For L between 2.8 (to match Bergal et al. 2022) and 22.6 (to match Schneider et al. 2007), the best fit of the model to the unfolding curve of (Lippok et al. 2016) is calculated. The shaded region in Fig. 5a shows the range in fitted behaviour as L varies, the fitting used in Sects. 3.1 and 3.3 is shown by the black line. For all L values, we are able to obtain a mean error within 2% of the (Lippok et al. 2016) curve in pure shear flow.

The predicted behaviour in pure elongational flow is shown in Fig. 5b. The shaded region represents the solution evaluated using the best fit of parameters from Fig. 5a. The predicted 50% unfolding threshold in pure elongational flow varies between approximately $\dot{\gamma }=1947$ s^-1^ and 3223 s^-1^. The smallest unfolding threshold of 1947 s^-1^ is obtained when the largest value of L$\,=22.6$ is used, showing that the proteins which are capable of sustaining very large extensions also unfold at lower shear rates. The significant variability in the pure elongational flow thresholds demonstrates a large degree of sensitivity in the model output to the value of L selected and further motivates the need to experimentally quantify the extension VWF is able to sustain in flow.

### VWF behaviour in idealised arterial flow

**Fig. 6:**
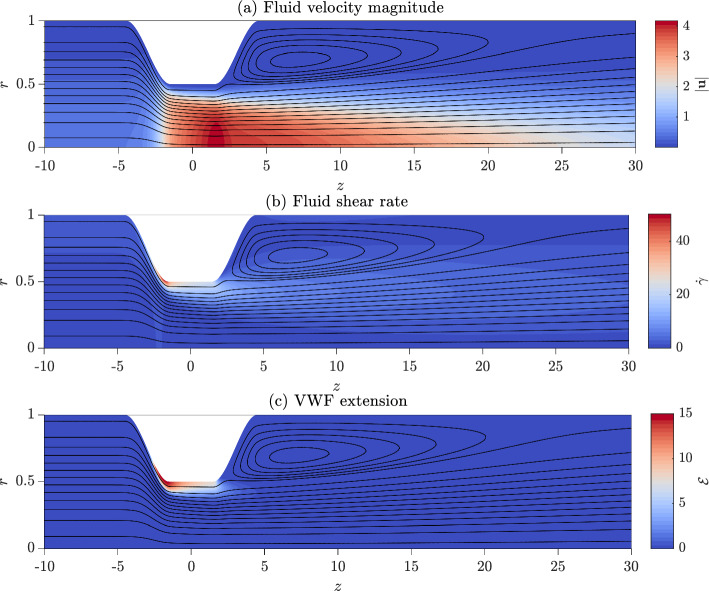
Dimensionless numerical solutions for $Re=400$ overlayed by fluid streamlines.**a** Fluid velocity magnitude. A recirculation zone forms downstream of the stenosis, indicated by circular streamlines. **b** Fluid shear rate, which is greatest at the leading edge of the stenosis. **c** VWF extension. The proteins are most extended by the stenosis wall and are fully extended at the leading edge of the stenosis. Parameter values: $\hat{l}_1=1.5,\ \hat{l}_2=2,\ \hat{h}=0.5$, L$\,=22.6.$

We now examine the model’s predictions for VWF’s behaviour in steady stenoic arterial flow. Figure 6 shows the dimensionless numerical solution of the model obtained for $Re=400$. All subfigures illustrate solutions overlaid by the fluid closed streamlines. At this Reynolds number, a recirculation zone forms downstream of the stenosis as illustrated by the streamlines. The magnitude of the fluid velocity is shown in Fig. 6a. The flow is four times faster as it crosses the stenosis compared to upstream. The fluid shear rate, shown in Fig. 6b, is greatest at the leading edge of the stenosis at $z=-2$ where it reaches $\dot{\gamma }\approx 55$. The shear rate is much lower away from the boundary and in the flow recirculation zone. VWF extension $\mathcal {E}$ is shown in Fig. 6c. VWF reaches $\mathcal {E}\approx 15$ which is the maximum extension achievable with a maximum VWF length of L$\,=22.6$. The maximum extension is obtained at the leading edge of the stenosis at $z=-2$ where $\dot{\gamma }$ is the greatest.

#### The effect of Reynolds number on VWF unfolding

We now examine how VWF extension changes as the Reynolds number varies for a fixed stenosis geometry. In this section to compare the shear rate obtained at the boundary for different flow rates, we define the scaled wall shear rate (WSR) as the dimensionless shear rate multiplied by the Reynolds number, $Re \dot{\gamma }$, this remains dimensionless but reflects how the magnitude of the dimensional shear rate changes as the flow rate increases.

**Fig. 7:**
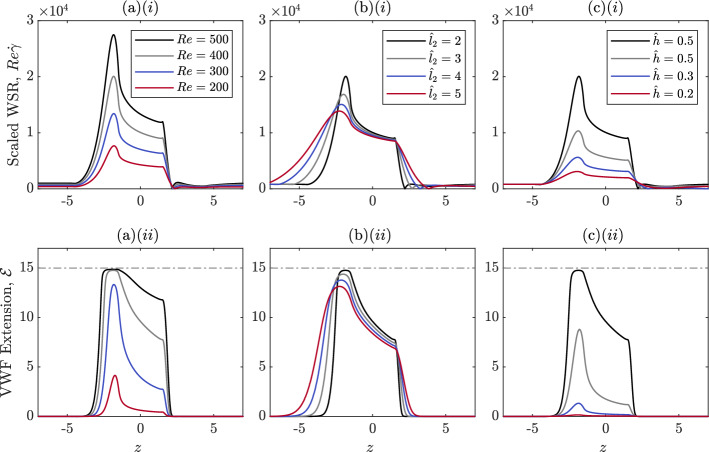
VWF extension for varying stenosis geometry and Reynolds numbers. When not stated in the legend, all other geometry parameters are: $\hat{l}_1=1.5$, $\hat{l}_2=2$, $\hat{h}=0.5$, L$\,=22.6$, and $Re=400$. Columns show behaviour for **a** increasing Re, **b** increasing stenosis steepness, and **c** increasing stenosis height respectively. Subfigures (*i*) show the scaled wall shear rate (WSR), defined as $Re\dot{\gamma }$, and the corresponding subfigures (*ii*) show the VWF extension obtained at the wall

The scaled wall shear rate on the stenosis wall for *Re* from ranging 200 to 500 is shown in Fig. 7a*i*, illustrating that as the Reynolds number increases, the shear rate increases. The maximum shear rate occurs at the leading edge of the stenosis for all Re. VWF extension on the stenosis wall is shown in Fig. 7a*ii*. For all Reynolds numbers, the maximum extension is obtained at the point on the stenosis wall where the wall shear rate is greatest. As Re increases, VWF extends more at the stenosis wall as a result of the increasing shear rate. Furthermore, the nonlinear dependence of VWF extension on the shear rate is demonstrated as the protein reaches an extension of nearly 100% at Re = 400 but only 33% at $Re=200$.

#### Stenosis geometry and VWF extension

In Sect. 3.3.1, we found that the greatest VWF extension is obtained at the wall; hence, we now examine how varying stenosis geometry alters the value and axial position of this extension for fixed Reynolds number of Re = 400. The scaled wall shear rate and VWF extension for increasing stenosis steepness are shown in Fig. 7b*i*, b*ii*. We increase the stenosis steepness by decreasing the parameter $\hat{l}_2$. Increasing the steepness of the stenosis slightly increases the maximum shear rate in the pipe, causing the VWF to unfold more. However, for steeper stenoses, the increased shear rates, and correspondingly VWF extension, are confined to a smaller region.

The wall shear rate and VWF extension for increasing stenosis height are shown in Fig. 7c*i*, c*ii*. Increasing the stenosis height drastically increases the maximum shear rate in the pipe and causes VWF to unfold to a greater extent. For smaller stenoses with $h\le 0.2$, a fluid recirculation zone does not form since the shear rate $\dot{\gamma }>0$ for all $z>0$. The absence of a recirculation zone means that there will be more significant transport of VWF behind the stenosis which could alter thrombus location.

#### Elongational flow structures in arteries and VWF unfolding

**Fig. 8:**
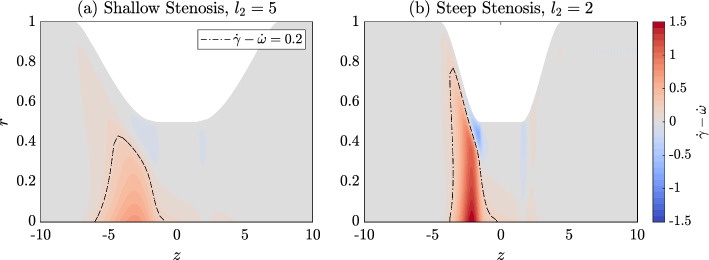
Flow structures over the stenosis. The flow is elongational when ${\dot{\gamma }}-{\dot{\omega }}\ll 0$ and rotational when ${\dot{\gamma }}-{\dot{\omega }}\ll 0$. Flow over: **a** a shallow stenosis with $\hat{l}_2=5$ and **b** a steeper stenosis with $\hat{l}_2=2$. For the two cases, the region where ${\dot{\gamma }}-{\dot{\omega }}=0.2$ is shown by the dashed black line, the interior defines regions in which we have significantly more elongation than rotation. The elongational flow (**b**) has a much larger disparity between the shear rate and the rotation rate at the entrance to the stenosis. Both have: $\hat{l}_1=1.5,\ \hat{h}$ = 0.5, and Re = 400

In Sect. 3.3.2, we showed that increasing the steepness of the stenosis alters the flow, leading to a higher wall shear rate. Figure 8 shows the difference between the shear rate and the rotation rate, ${\dot{\gamma }}-\dot{\omega }$, for a steep stenosis compared to a more shallow stenosis, with red regions on Fig. 8 showing regions of elongational flow and blue regions showing rotational flow. The steeper stenosis leads to elongational flows with ${\dot{\gamma }}-\dot{\omega }$ three times larger than the shallow stenosis.

To highlight this, we show regions for which ${\dot{\gamma }}-\dot{\omega }=0.2$, by the dashed regions in Fig. 8. The interior of this line defines regions where the flow is highly elongational. The maximum shear rate obtained in these highly elongational regions is ${\dot{\gamma }}=1.3$ and ${\dot{\gamma }}=3.7$ for the shallow stenosis and steep stenosis, respectively. These correspond to ${\dot{\gamma }}=317.1\,\text {s}^{-1}$ and ${\dot{\gamma }}=902.0\,\text {s}^{-1}$ in dimensional terms. For L$\,=22.6$, our model predicted that in pure elongational flow, VWF is half unfolded at $1947\,\text {s}^{-1}$ whereas VWF is half unfolded at $5096\,\text {s}^{-1}$ in pure shear flow. Since the flow in the centre region of the stenotic artery is not pure elongational flow, we expect that the unfolding threshold in this region will be larger than $1947\,\text {s}^{-1}$ but still smaller than the pure shear flow unfolding threshold. In the highly elongational regions, the shear rate does not reach the pure elongational flow threshold of $1947\,\text {s}^{-1}$. As a result, VWF only unfolds to 2.3% and 0.7% of its maximum length in the indicated regions in Fig. 1. This is in contrast with the extension achieved at the wall where VWF can reach extensions of 98% and 88% in the steep and shallow stenosis cases, respectively. The lack of significant unfolding in the elongational flow region is in contrast with the work of Zhussupbekov et al. (2021) where the authors found that VWF will be fully extended in the centre of the flow.

The predicted significance of elongational flow on VWF unfolding depends on model parameterisation and the predicted unfolding rate in pure elongational flow. In Sect. 3.2, we demonstrated that our predicted unfolding thresholds in pure elongational flow range from approximately 2000–3200$\,\text {s}^{-1}$ depending on the maximum VWF length L, which is not known. The smallest unfolding threshold was found for proteins with the largest maximum lengths L$\,=22.6$ for which we did not find significant unfolding away from the walls. However, these quantitative predictions are subject to change as more experimental insights into VWF length and unfolding behaviour become available.

## Discussion

In this paper, we have presented a model for the dynamics of shear-sensitive blood protein VWF using a dilute limit of the viscoelastic fluid model FENE-P with a modified relaxation time. The modified relaxation time captures VWF propensity to unfold in response to the fluid shear rate. This is characterised using parameter estimates from the VWF unfolding behaviour measured by Lippok et al. (2016). Through a configuration tensor, our model can describe VWF’s length and orientation in any combination of elongational, shear, and rotational flows, defined as $\dot{\gamma }\gg \dot{\omega }$, $\dot{\gamma }\approx \dot{\omega }$, and $\dot{\gamma }\ll \dot{\omega }$, respectively. Using an idealised arterial stenosis geometry, we demonstrated that increasing the fluid flow rate and stenosis height have the strongest effect on the wall shear rate and therefore VWF’s extension at the wall. Since platelets are transported in large quantities in the cell-free layer by the wall, VWF molecules which are extended close to the arterial wall will most readily bind with platelets to form a thrombus Casa and Ku (2017).

Our model is able to reproduce the dependency of VWF behaviour on the flow structure, namely that the protein unfolds at lower shear rate in pure elongational compared to pure shear flow (Babcock et al. 2003; Smith et al. 1999). Our prediction of the shear rate at which VWF unfolds in pure elongational flow varies depending on the value of maximum extension which VWF can achieve, which is not definitively established in the literature. In our model, the parameter L restricts the value of VWF extension. For L$\,=2.8$–22.6, VWF can reach at most $\approx 2--15$ times its natural length. For this range of the maximum VWF length, we estimate that VWF will be 50% unfolded between approximately 2000–$3200\,\text {s}^{-1}$ in pure elongational flow. This agrees with existing discrete models of VWF which uniformly estimate that VWF unfolds at a lower shear rate in elongational flow compared to shear flow. Furthermore, our estimated range of the unfolding threshold in pure elongational flow falls within the range of values predicted by discrete mathematical models of single VWF molecules: 500$\,\text {s}^{-1}$ (Sing and Alexander-Katz 2010), 2400$\,\text {s}^{-1}$ (Nguyen et al. 2021), 2500$\,\text {s}^{-1}$ (Kania et al. 2021), and 3500 $\,\text {s}^{-1}$ (Dong et al. 2019).

This model is able to examine VWF behaviour in the complex, multidimensional flows which occur in diseased arteries. We show that VWF is most unfolded in the shear flow close to the stenosis wall, with the maximum extension occurring at the leading edge of the stenosis. This provides patterns of elongation of the protein along the wall which could be combined with a model of platelet transport to predict thrombus formation. We have shown that elongational flow occurs within stenosed geometries, with the difference between the shear rate and the rotation rate increasing as the steepness of the stenosis increases. Our model can evaluate the degree to which VWF unfolds in free flow away from the wall compared to the wall extension. Using a single value of VWF extension which matches the data of Schneider et al. (2007), namely L$\,=22.6$, our model predicts VWF only reaches 2% of its maximum length in the highly elongational flows away from the wall where the maximum shear rate is 171$\,\text {s}^{-1}$. However, our parameter sensitivity analysis demonstrated that the model predictions in elongation flow are sensitive to the maximum length of VWF used. Furthermore, we fixed the unfolding mechanics of our model to fit the unfolding curve of Lippok et al. (2016), it is possible that if further sources of experimental data are incorporated, there could be some parameter regimes, in which significant unfolding could be found away from the wall.

The structure of our model differs from the only continuum model of VWF to date by Zhussupbekov et al. (2021). Zhussupbekov et al. (2021) uses experimental data from DNA unfolding to define regions where the flow is sufficiently elongational to unfold VWF (Babcock et al. 2003). Zhussupbekov et al. (2021) then enforce that the proteins unfold at 500$\,\text {s}^{-1}$ in these regions of elongational flow. Using these parameter choices (Zhussupbekov et al. 2021) predict that VWF will fully unfold in the flow away from the wall in microfluidic stenosis simulations. Our model does not include a threshold at which the flow is classified as elongation; instead, the *flow structure is encoded *in (5) through the velocity gradient. The velocity gradient is then combined with a single constitutive relaxation time which models VWF’s ability to unfold. This allows our model to be easily parameterised using data from shear flow, eliminating the need to rely on data obtained for other proteins, which may not be accurate for VWF.

The accuracy of our predictions relies on the estimation of the model parameters which describe VWF’s unfolding through the nonlinear relaxation time $\tau$. We estimated these parameters, aside from VWF length *L*, by comparing our model predictions in shear flow to the unfolding behaviour measured in Lippok et al. (2016). This required the estimation of five unknown parameters. Our estimate yields a 1.82% error in the relative length of VWF compared to (Lippok et al. 2016). However, this estimation was done using a single minimsation algorithm, and it is possible that alternative minima could exist which yield a better fit to the Lippok et al. curve. Finally, in this paper, we varied the maximum VWF length to determine the variation in best fit obtained to the Lippok et al. (2016) data. The resulting predicted behaviour in pure elongational flow varied significantly over the range of L = 2.8–22.6. When further data are available for the maximum extension of VWF in free flow, the model parameters which determine VWF unfolding can be readily updated allowing the model to more precisely estimate the elongational flow behaviour of VWF.

There are two different hypotheses about VWF’s microscale mechanics, namely whether it behaves like a collapsed polymer (Schneider et al. 2007) or a loosely coiled polymer (Bergal et al. 2022). In this paper, we estimated our model’s parameters using the unfolding curve of Lippok et al. (2016), which was obtained using a *discrete numerical* collapsed polymer model fit to experimental VWF cleavage data. However, both of the microscale behaviour hypotheses of VWF can be captured by the modified FENE-P model in this paper. Our model is derived from the microscale mechanics of a two-bead dumbbell; therefore, the complexity of VWF’s unfolding is captured in the nonlinear relaxation time function. Collapsed and loosely coiled polymers both reach a maximum length as the force increases and obtain a nonlinear force–extension curve, both of which are features of our model; therefore, our model can be adapted as more evidence appears to support either hypothesis.

There are several limitations and possible extensions of the theoretical framework of our model which we now detail. Firstly, our model does not include any history effects, for instance, the proteins do not require exposure to high shear stresses for a certain period of time to unfold. Furthermore, our model does not include the hysteresis of VWF, whereby the proteins relax back to their original length over a longer timescale than extension. This would mean that the proteins could remain unfolded downstream of the stenosis which could be significant for thrombus formation behind the stenosis. Including hysteresis would allow the model to be applied to the pulsatile flow which occurs in vivo in arteries. Pulsatile flow within stenosed pipes alters the recirculation zone within each cardiac cycle and creates time-varying vortices (Sherwin and Blackburn 2005). The time of an average cardiac cycle in the coronary artery is approximately 0.8 s, which is much longer than the timescale required to unfold VWF (Chen et al. 2021). Our model currently enforces that VWF refolds rapidly, this means that it will underestimate the protein’s length over the cardiac cycle and predicts that the proteins are not extended the recirculation zone. In reality, the proteins could remain extended during the cardiac cycle due to their slow refolding time. Accurately capturing the time-dependent dynamics of VWF length throughout the domain would be especially important when combined with the additional mixing from pulsatility. Our model could be extended to include hysteresis by following the construction in Zhussupbekov et al. (2021) and categorising the proteins as extending, which unfold rapidly, and retracting, which refold more slowly. However, this would require formulating how proteins move between the two categories, adding significant complexity to the model in physiological flows.

There are several theoretical extensions to our modelling framework which would improve its ability to describe VWF when in close proximity to the walls of an artery or those of an in vitro device. In this paper, we used the solution of the FENE-P equation to describe VWF length when at the vessel wall. However, the FENE-P equation is derived for a protein in the absence of walls. The effect of walls has been included in similar non-Newtonian models of confined flows of proteins (Biller and Petruccione 1987) and confined flow of bacteria (Saintillan and Shelley 2015). However, this introduces reflection conditions or binding conditions on the probability density function from which the configuration tensor is derived. This adds complexity to the model construction as the arising equation for the configuration tensor does not have a closed form (Biller and Petruccione 1987). VWF unfolding behaviour when tethered to a non-reactive wall differs significantly from its behaviour in free flow, so it is not clear if the unfolding relation fitted in shear flow used in this paper would effectively describe the dynamics of VWF when close to or bound to a wall (Fu et al. 2017). Furthermore, in this paper to improve the tractability of numerical simulations, we added numerical diffusion to the FENE-P equation. This addition necessitates the selection of boundary conditions for the FENE-P equation on the walls of the device. In this paper, we imposed no diffusive flux boundary conditions on the device walls, this choice offers ease of implementation in the Finite Element Method and is well-suited to flow setups where the walls are solid, rigid, and non-reactive. This choice must be carefully examined when using the model to examine VWF dynamics in more complex devices; for instance, is it not clear what the best choice of boundary condition would be for a porous boundary or one where VWF is able to bind to the walls. It would be especially valuable to characterise the appropriate boundary conditions for materials commonly used in medical and experimental devices such as polydimethylsiloxane (PDMS) (Berry et al. 2021). Indeed, when binding to a collagen-coated wall, VWF has been shown to form bundles or carpets of tangled proteins (Colace and Diamond 2013; Schneider et al. 2007); since the FENE-P equation describes dilute suspensions of polymers or proteins, our model would not be able to capture this binding or protein–protein interactions. Insights from discrete models of VWF could be used to effectively determine how best to include the effects of binding or protein–protein interactions into a continuum framework (Liu et al. 2022; Wang et al. 2019).

In this paper, we examine flow and VWF dynamics within arterial scale stenoses, as this is the most clinically relevant scale and geometry at which high shear thrombosis occurs. However, VWF-mediated thrombosis can also occur at the location of an arterial stent or on a prosthetic heart valve (Casa and Ku 2017). Our model can be readily applied to examine these alternative geometries or indeed any vessels or devices in which the continuum approximation for the VWF suspended in blood is valid. This holds when the vessel diameter is significantly larger than the radius of a red blood cell (approximately 3.5 μm (Colace and Diamond 2013)). As a result, our model can be applied in smaller vessels such as arterioles or in microfluidic devices which are regularly used to study thrombosis *in vitro* (Liu et al. 2022; Westein et al. 2013).

## Conclusion

In this paper, we have presented a novel continuum model to describe the dynamics of VWF in blood. Our model uses a single constitutive relation to describe VWF’s propensity to unfold at a given shear rate which is parameterised to match experimentally measured VWF behaviour in shear flow. The model is then able to quantitatively predict VWF length and orientation in any combination of flow types which occur in diseased arteries. Crucially, our model can examine VWF dynamics in elongational flows which are challenging to examine experimentally and which are predicted to facilitate excessive VWF unfolding. Our model could be readily incorporated into a continuum model of high shear thrombosis by coupling the configuration tensor to an advection–diffusion equation which tracks the concentration of VWF in the flow. The combination of VWF concentration and the configuration tensor can then be used to determine in which regions VWF will be both in high concentrations and unfolded, and therefore, where thrombosis could occur.

## Figures and Tables

**Fig. 9:**
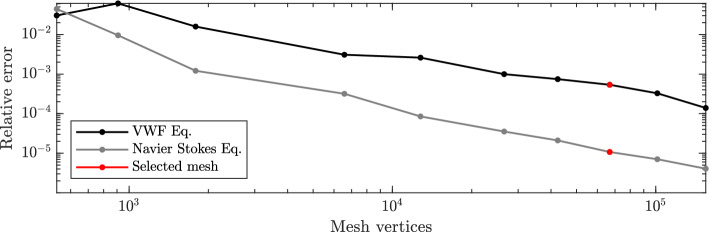
**a** Relative error in maximum extension of the FENE-P equation and maximum fluid velocity for increasing numbers of mesh vertices. **b** Convergence of the minimum value of VWF extension to zero. Selected mesh shown in red. Parameters: $\hat{h}=0.5, \hat{l}_1=1.5,\ \hat{l}_2=2$, Re=500, L$\,=22.6$

## Data Availability

Files for numerical solution and figure production can be found in the repository https://github.com/Edwina-Yeo/VWF-Modelling.

## Appendix A: Parameter estimation

We estimate the model parameters which determine VWF’s unfolding, namely $\alpha$, $\beta$, $\delta$, and $\dot{\gamma }^*$, using the VWF cleavage rate of Lippok et al. (2016) normalised using the maximum cleavage rate obtained in their work ($3.5 \times 10^{-3}$nM/s). Following the assumption of Lippok et al. (2016) that the proteins cleave at a rate proportional to their length, this normalised cleavage rate represents the normalised VWF extension. We use the solution of the FENE-P equation in two-dimensional shear flow at $\dot{\gamma }_i$, which we denote as $\varvec{A}(\dot{\gamma }_i)$ to calculate the extension at that shear rate $\mathcal {E}(\dot{\gamma }_i)$ using (4). We use this extension to define the mean error made to Lippok et al. (2016) curve as

A1 $$\begin{aligned} E=\frac{1}{N}\sum _{i=1}^{N}|\tilde{\mathcal {E}}(\dot{\gamma }_i)- \mathcal {E}(\dot{\gamma }_i)|, \end{aligned}$$

where in practice, we use $N=400$ discrete values of the shear rate between $\dot{\gamma }=1\,\text {s}^{-1}$ and $\dot{\gamma }=10^5\,\text {s}^{-1}$ equally spaced on a log scale. We use the gradient-based minimiser *fmincon* from MATLAB’s optimisation toolbox to determine the VWF parameters which minimise *E*. We use the initial guess of $\beta =0.01\,\text {s}$, $\dot{\gamma }=10^4\,\text {s}^{-1}$, $\alpha =0.01\,\text {s}$, and $\delta =10^{-4}$.

We first seek a set of optimal parameters with the maximum VWF length fixed at L$\,=22.6$ which is set so that the maximum extension VWF can achieve in two-dimensions matches the maximum extension measured by (Schneider et al. 2007). We set bounds on the minimisation so that we seek optimal parameters which satisfy $10^{-8}<\delta <10^{-3}$, $10^{-8}\,\text {s}<\beta <10^{-2}\,\text {s}$, $10^{-6}\,\text {s}<\alpha <0.1\,\text {s}$, $4000\,\text {s}^{-1}<\dot{\gamma }^*<2\times 10^4\,\text {s}^{-1}$, which are motivated by the physical role of each parameter in the relaxation time. We find that the parameter values listed in Table 1 minimise the error obtained compared to (Lippok et al. 2016) with $E= 0.0182$ as defined in (A1), which is equivalent to an average percentage error of 1.82%.

In Sect. 3.2, we vary the value of L used. We use five values of L between 2.8 and 22.6, obtaining a best estimate of the parameters: $\alpha$, $\beta$, $\delta$ and $\dot{\gamma }^*$, in each case using numerical continuation. We keep the bounds on the parameter space unchanged in this process.

## Appendix B: Numerical scheme and validation

We use the package FEniCS version *2019.2.0.dev0*, and code construction is based on examples in Alnæs et al. (2015); Logg et al. (2012) for the solution of Stokes equations and advection–diffusion equations.

In our numerical solution of the steady Navier–Stokes equations (8), we use Taylor Hood elements of the first- and second-order for the pressure and velocity vector, respectively. The steady nonlinear system is solved using the inbuilt Newton Solver *solve* as part of the FEniCS package. We solved the model using the viscous pressure scale which is equivalent to solving for the dimensionless pressure defined in (7) divided by the Reynolds number. The velocity gradients in each direction, along with the wall shear rate on the pipe wall, are determined using the first-order elements as functions of the velocity solution. The velocity gradients and velocity field are then used to solve the modified FENE-P Eq. (9).

The FENE-P equation consists of four coupled advection–diffusion equations with the velocity field given by the solution of the Navier–Stokes equations. As discussed above, we include diffusion in these equations for numerical tractability. We use the first-order Lagrange elements to solve for each component of the configuration tensor. To solve the system for each component of the configuration tensor, $\varvec{A}$, we employ continuation in the Reynolds number, with unit steps performing well. Our initial guess for the solver for $Re=0$ is that $\varvec{A}=\varvec{I}$. The inlet value of the configuration tensor is found by solving Eq. (9) under the imposed inlet flow for which we solve the nonlinear system numerically, using the *NumPy* Newton solver *fsolve* without numerical continuation, with an initial guess of $\varvec{A}=\varvec{I}$.

We construct a mesh in GMSH which is finer closer to the boundary and at the upstream edge of the stenosis where the shear rate is greatest. In Fig. 9a, we compare the maximum value of VWF extension and fluid velocity magnitude obtained on a sequence of meshes with increasing numbers of mesh vertices to the same quantities obtained using a fine reference mesh with $6\times 10^5$ vertices. We use the mesh that has approximately $6.7\times 10^4$ vertices on which we achieve a maximum relative error of 0.1% on all variables compared to the aforementioned reference mesh. We define the relative error by dividing the difference between the value on the fine reference mesh and the coarse mesh by the maximum absolute value on the fine mesh.

The steep gradients in the fluid shear rate can lead to spurious oscillations in the components of the configuration tensor $\varvec{A}$. To ensure that our mesh is suitably fine to capture the large gradients in the tensor components without oscillations, we examine the minimum value of the trace of the configuration tensor obtained on each mesh. The trace $\varvec{A}$ should be bounded below by one. Our selected mesh gives a relative error, defined as $(Tr(\varvec{A})-1)/(L^2-1)$, equal to $-$ 0.0022; hence, the amplitude of any oscillations in the configuration tensor components is less than 1% when compared to the maximum extension.
